# Values of hemodynamic variation in response to passive leg raising in predicting exercise capacity of heart failure with preserved ejection fraction

**DOI:** 10.1097/MD.0000000000005322

**Published:** 2016-11-04

**Authors:** Hong-Lian Zhou, Ling Ding, Tao Mi, Kai Zheng, Xiao-Fen Wu, Jing Wang, Meng-Ying Liu, Le Zhang, Cun-Tai Zhang, Xiao-Qing Quan

**Affiliations:** Department of Geriatrics, Tongji Hospital, Tongji Medical College, Huazhong University of Science and Technology, Wuhan, China.

**Keywords:** 6-minute walk distance, exercise capacity, heart failure with preserved ejection fraction, impedance cardiography, passive leg raising

## Abstract

In heart failure patients with preserved ejection fraction, their hemodynamic parameters usually change when they are from recumbent to passive leg raising. The authors designed this study to investigate the relationship between hemodynamic parameters measured by impedance cardiography (ICG) and 6-minute walk distance (6MWD) of heart failure with preserved ejection fraction (HFPEF). We recruited 49 subjects with HFPEF in the study, and all the subjects were separated into 2 groups: the patients whose hemodynamic parameters rose after passive leg raising were in group 1 (n = 26) and the patients whose hemodynamic parameters did not rise after passive leg raising were in group 2 (n = 23). Our study then compared the 6MWD, left ventricular ejection fraction, and plasma NT-pro-brain natriuretic peptide between the 2 groups. Group 1 had significantly longer 6MWD than group 2 (515.38 ± 24.97 vs 306.39 ± 20.20 m; *P* = 0.043). Hemodynamic parameters measured by ICG significantly correlated with 6MWD in both groups. Patients whose hemodynamic parameters rose in response to passive leg raising were more likely to have better exercise capacity. Hemodynamic variation in response to passive leg raising measured by ICG may be more sensitive in predicting exercise capacity of patients with HFPEF.

## 1 Introduction

Heart failure with preserved ejection fraction (HFPEF) has been regarded as a clinical entity distinct from other forms of heart failure and defined predominantly by symptoms of dyspnea and fluid retention in the absence of a significant reduction in left ventricular (LV) systolic function.^[1,2]^ The prevalence of HFPEF has been increasing, and the morbidity, mortality, and healthcare costs has been equal to heart failure with reduced ejection fraction.^[3]^ Knowledge of a patient's actual cardiac function is important for the treatment of HFPEF.^[4]^ Predicting exercise capacity and severity of cardiac dysfunction of patients with HFPEF may contribute to better management of HFPEF.

Passive leg raising (PLR) may identify patients with impairment of diastolic functional reserve during exercise.^[5]^ PLR is a reversible fluid-loading maneuver,^[6]^ which may potentially increase intrathoracic blood volume, cardiac preload, and then cardiac output (CO), through circulating venous blood from the legs^[7]^ towards the thorax.^[8]^ CO increased in healthy persons in response to PLR.^[9]^ For patients with impaired cardiac function, 1 of compensatory mechanisms to maintain normal CO is the Frank–Starling mechanism. The Frank–Starling mechanism states that an increase in diastolic filling causes an increase in peak systolic atrial pressure,^[10]^ representing the intrinsic capability of the heart to respond to enhance preload with an increase in force development.^[11]^ Cardiovascular responses to PLR is useful in assessing preload reserve, but it has seldom been studied longitudinally in predicting severity of cardiac dysfunction in HFPEF.

Impedance cardiography (ICG), a reliable and noninvasive technique, can be used to measure hemodynamic parameters continually.^[12,13]^ The fundamental of ICG is Ohm law, which states that a constant current travels through a conductor as a result of voltage change directly proportional to variations in impedance.^[12,14]^ A considerable proportion of previous data have confirmed the role of hemodynamic parameters measured by ICG in estimating cardiac function.^[15–17]^ The hemodynamic parameters CO, cardiac index (CI), stroke volume (SV), stroke volume index (SVI), left stroke work (LSW), and left stroke work index (LSWI) correlated positively with cardiac function.^[18]^ Stroke systemic vascular resistance (SSVR) represents the resistance of blood flow in the vascular system. Stroke systemic vascular resistance index (SSVRI) is the systemic vascular resistance normalized for body surface area. Both variables reflect the afterload of the heart and the degree of arteriosclerosis in the systemic artery.^[19]^

The present study will research the correlation among 6-minute walk distance (6MWD), left ventricular ejection fraction (LVEF), plasma NT-pro-brain natriuretic peptide (NT-proBNP), and hemodynamic parameters in patients with HFPEF. Our study will further explore the values of hemodynamic variation in response to PLR in predicting exercise capacity of patients with HFPEF.

## 2 Methods

### 2.1 Patients and controls

This observational study was approved by the Ethics Committee of Tongji Hospital, Wuhan, China. All the subjects had received informed consent and signed in informed consent before enrollment. The study was performed from January 2014 to June 2016. Our study recruited patients with HFPEF, and the inclusion criteria in our study were based on the following: typical symptoms of heart failure; representative signs of heart failure; the LVEF ≥50% (by echocardiography); evidence of diastolic dysfunction on echocardiography (mitral inflow E/A ratio, E′ measured at the mitral annulus, and E/E′ ratio).^[20,21]^ Subjects with impaired cognition, atrial fibrillation, chronic obstructive pulmonary disease (COPD), asthma, severe hepatic disease, severe renal impairment, hyperthyroidism, arthritis, ankle, knee or hip injuries, and muscle wasting were excluded.^[22,23]^ We even did not recruit patients with systolic blood pressure (SBP) of more than 180 mm Hg, or diastolic blood pressure (DBP) of more than 100 mm Hg or resting heart rate of more than 120, drugs and/or alcohol abuse, or any life-threatening disease.^[22,23]^Additionally, we excluded patients with recent myocardial infarction, unstable angina, pacemaker implantation, enlarged LV dimension, candidacy for revascularization, cardiomyopathy, left atrial enlargement, and valvular heart disease.

“Responds to PLR” in our study meant that CO calculated by ICG was changed in participants when they were from supine position to PLR. All the subjects were separated into 2 groups according to CO variation in response to PLR: the patients whose CO increased in response to PLR were in group 1 (n = 26), and the patients whose CO did not increase in response to PLR were in group 2 (n = 23). The 2 groups were matched for age, sex, height, weight, body mass index (BMI), underlying disease (chronic renal disease, coronary artery disease, diabetes mellitus, hypertension), and basic medicine [β-receptor blocker, digoxin, angiotensin-converting enzyme inhibitor (ACEI), angiotensin receptor blocker (ARB)].

### 2.2 Clinical evaluation

**Figure 1:**
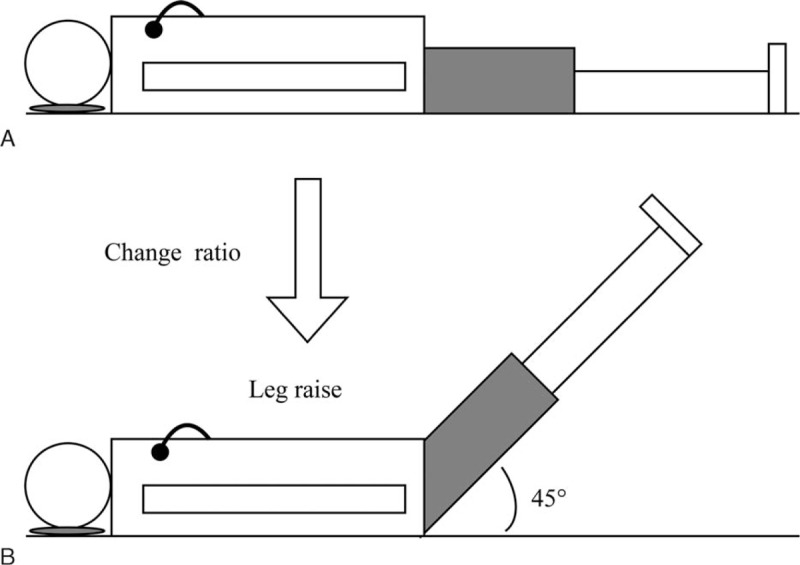
A, The subject was put electrodes on the neck and hypochondriac region and performed ICG when he was in the supine position. B, The subject was put electrodes on the neck and hypochondriac region and performed ICG when he was raising legs at 45°. ICG = impedance cardiography.

The machine to perform ICG was the Cheer Sails Medical (CSM3000 system). The basic principle of ICG was that specific waveform that can be used to calculate SV appeared as a result of the impedance changing with high-frequency (75 kHz) and low magnitude (1.8 mA) current across the thorax during cardiac ejection.^[17,23]^ After 5 minutes’ rest, the technician put electrodes on the neck and hypochondriac regions of patients and performed ICG for 3 minutes when the subjects were in the supine position (Fig. 1A). After 30 minutes of rest, the technician performed ICG again for the same subjects for 3 minutes when they were raising legs at 45° (Fig. 1B).

The 6MWT is used to measure the distance that a patient can quickly walk on a flat and hard surface in 6 minutes.^[24]^ 6MWT was ordered by the physicians as an initial or follow-up assessment in cardiac function according to ATS standards.^[13,14]^ We obtained the reference standards for the distance walk during the 6MWT from the study by Enright and Sherrill.^[23]^ All distance of 6MWT were recorded as 6MWD accurately.

Weight and height were recorded to calculate BMI values. Our study also recorded underlying diseases (chronic renal disease, coronary artery disease, diabetes mellitus, and hypertension) or basic medicine (β-receptor blocker, digoxin, ACEI, and ARB). NT-proBNP was measured using an automatic biochemistry analyzer.^[25]^ Our study measured LVEF and other parameters of cardiac structure by echocardiography, as previously reported.^[26–29]^

### 2.3 Statistical analysis

Analyses were carried out using IBM SPSS 16.0 for Windows (IBM, NY). Continuous variables were compared by independent-samples *t* test and dichotomous variables were compared by Pearson chi-square test in baseline subject characteristics ^[30]^. The means ± standard error of continuous variable was considered as statistic value.^[31]^ Dichotomous variables with fewer than 5 participants in a category were given continuity correction in chi-square test. Bivariate correlate analysis was used to compare the correlation among hemodynamic parameters, plasma NT-proBNP, and 6MWD. Sample size was calculated as per the following formula: n = 2 × [(u_α_ + u_β_) × σ/δ]^2^. The ratio of sample sizes was ≈1. ‘σ’ represented the population standard deviation of 6MWD. ‘δ’ was the difference in means of 6MWD in population between group 1 and group 2. Thus, the sample size in each group: n = 2 × [(u_α_ + u_β_) × σ/δ]^2^ = 21. All statistical tests were 2-sided. *P* < 0.05 was considered statistically significant.

## 3 Results

Baseline characteristics of group 1 and group 2 are presented in Table 1. The subjects in the 2 groups had no statistical difference in age, sex, height, weight, BMI, SBP, DBP, and mean arterial pressure (MAP). There was no statistical difference in comorbidity such as chronic renal disease, coronary artery disease, diabetes mellitus, and hypertension. There were 5 (19.2%) individuals in group 1, and 1 (4.3%) individual in group 2 using β-receptor blocker. The number of characters taking digoxin was 1 (3.8%) in group 1, and 1 (4.3%) in group 2 in our study. There were 3 (11.5%) individuals in group 1 and 2 (8.7%) individuals in group 2 using ACEI/ARB. The basic medicine such as β-receptor blocker (*P* = 0.125), digoxin (*P* = 0.724), and ACEI/ARB (*P* = 0.560) in the 2 groups had no statistical difference.

**Table 1:**
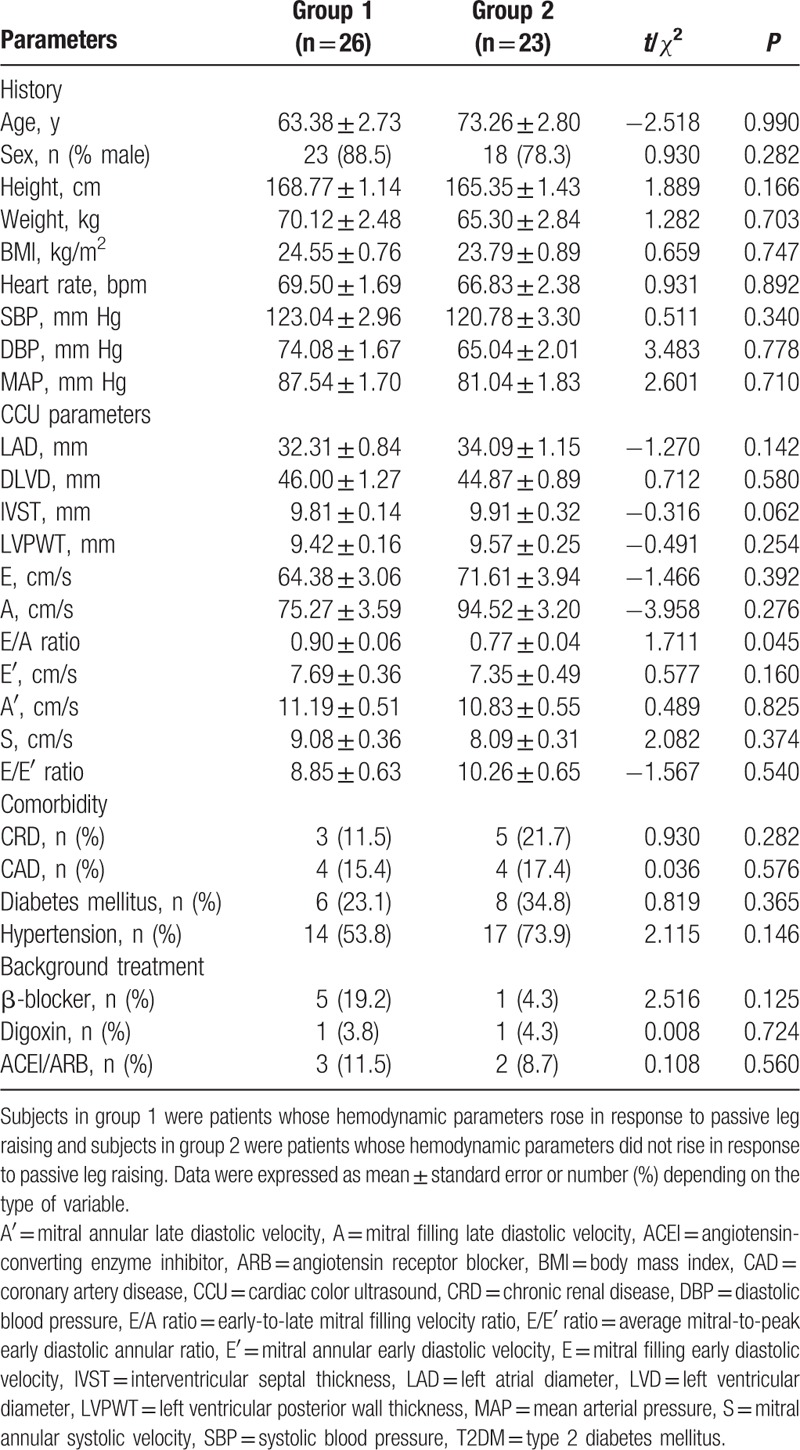
Comparisons of the clinical data between two group 1 and group 2.

There were no significant differences in most cardiac color ultrasound parameters (Table 1), for instance, left atrial diameter (LAD), diastolic left ventricular diameter (DLVD), interventricular septal thickness (IVST), left ventricular posterior wall thickness (LVPWT), mitral filling early diastolic velocity (E), mitral annular early diastolic velocity (E′), mitral annular late diastolic velocity (A′), mitral annular systolic velocity (S), average mitral-to-peak early diastolic annular ratio (E/E′) (8.85 ± 0.63 vs 10.26 ± 0.65; *P* = 0.540). But the early-to-late mitral filling velocity ratio (E/A) (0.90 ± 0.06 vs 0.77 ± 0.04; *P* = 0.045) in 2 groups were statistically different.

Table 2 shows the comparisons of hemodynamic parameters in group 1 when subjects were recumbent and PLR. In group 1, the parameters CO (3.70 ± 0.25 vs 4.15 ± 0.28 L/min), CI (2.04 ± 0.13 vs 2.28 ± 0.15 L/min/m^2^), SV (54.38 ± 3.68 vs 60.14 ± 4.02 mL), SVI (29.92 ± 1.85 vs 33.04 ± 2.01 mL/m^2^), LSW (61.77 ± 4.52 vs 68.69 ± 5.16 gm-m/beat), and LSWI (33.87 ± 2.23 vs 37.62 ± 2.52 gm-m/m^2^/beat) in recumbent were lower than those parameters in PLR. Whereas the parameters SSVR (444.75 ± 30.22 vs 407.36 ± 29.17 dynes/cm^5^) and SSVRI (247.94 ± 17.78 vs 226.87 ± 16.68 dynes/cm^5^/m^2^) in recumbent were higher than those parameters in PLR.

**Table 2:**
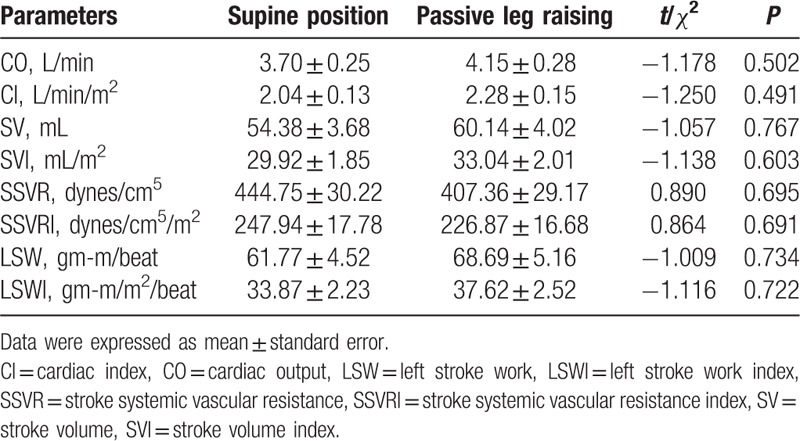
Comparisons of hemodynamic parameters in group 1 (n = 26).

Table 3 shows the comparisons of hemodynamic parameters in group 2 when patients were recumbent and PLR. In group 2, the parameters CO (3.14 ± 0.27 vs 2.93 ± 0.25 L/min), CI (1.82 ± 0.14 vs 1.69 ± 0.13 L/min/m^2^), SV (49.70 ± 4.64 vs 46.88 ± 4.10 mL), SVI (28.70 ± 2.37 vs 27.09 ± 2.10 mL/m^2^), LSW (52.49 ± 4.94 vs 50.03 ± 4.66 gm-m/beat), and LSWI (30.13 ± 2.47 vs 28.72 ± 2.34 gm-m/m^2^/beat) in recumbent were not lower than those parameters in PLR. Whereas the parameters SSVR (434.69 ± 43.64 vs 445.58 ± 39.83 dynes/cm^5^) and SSVRI (252.81 ± 23.60 vs 260.18 ± 22.17 dynes/cm^5^/m^2^) in recumbent were lower than those parameters in PLR.

**Table 3:**
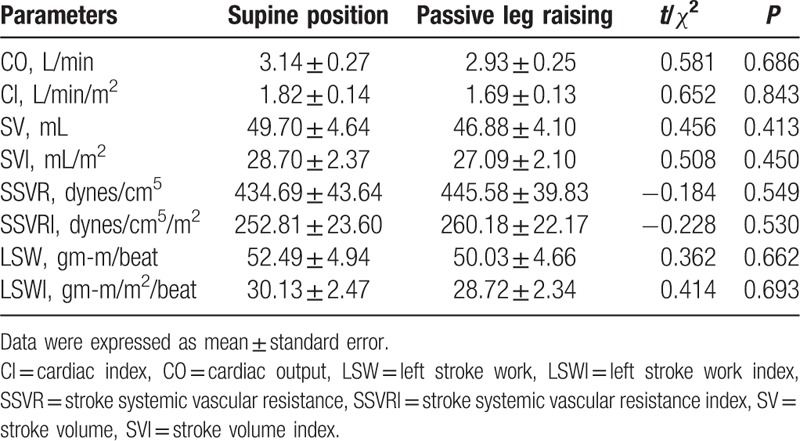
Comparisons of hemodynamic parameters in group 2 (n = 23).

The correlation among hemodynamic parameters, NT-proBNP, and 6MWD were showed in Table 4 and Table 5. When participants were at supine position, Pearson correlation analysis revealed that the parameters CO (*R* = 0.557, *P* < 0.001), CI (*R* = 0.493, *P* < 0.001), SV (*R* = 0.435, *P* = 0.002), SVI (*R* = 0.368, *P* = 0.009), LSW (*R* = 0.488, *P* < 0.001), and LSWI (*R* = 0.449, *P* < 0.001) significantly positively correlated with 6MWD. The parameters SSVR (*R* = −0.223, *P* = 0.124) and SSVRI (*R* = −0.312, *P* = 0.029) correlated inversely with 6MWD in the same group. The correlation among hemodynamic parameters and 6MWD was similar when patients were raising the leg. As shown in Table 5, NT-proBNP was statistically inversely correlated with 6MWD (*R* = −0.539, *P* < 0.001).

**Table 4:**
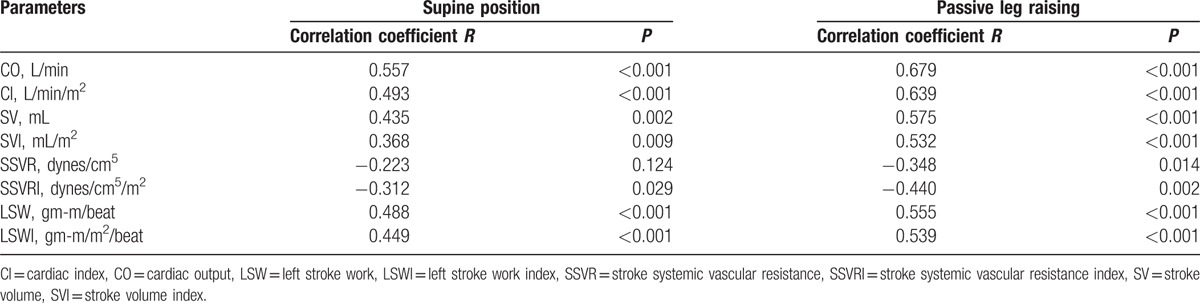
Correlation among hemodynamic parameters and 6MWD.

**Table 5:**
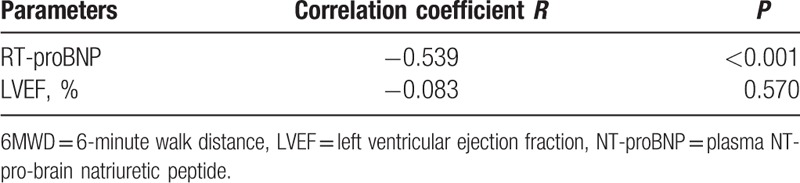
Correlation among RT-proBNP, LVEF, and 6MWD.

**Figure 2:**
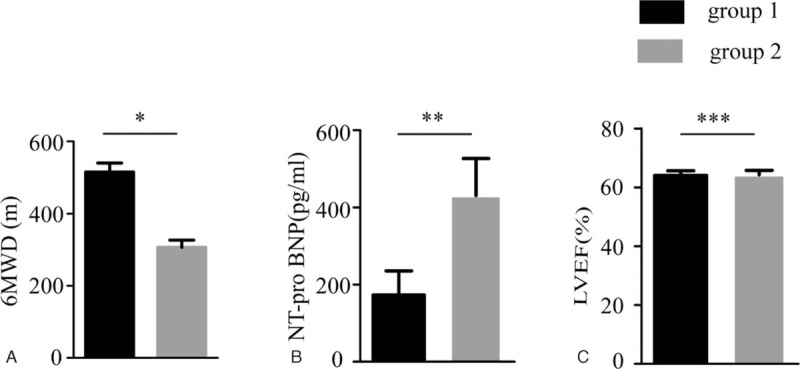
Comparisons of 6MWD, NT-proBNP, and LVEF when patients were supine in position. A, The comparison of the 6MWD between group 1 and group 2 (^∗^*P* = 0.043). B, The comparison of the NT-proBNP between group 1 and group 2 (^∗∗^*P* = 0.059). C, The comparison of the LVEF between group 1 and group 2 (^∗∗∗^*P* = 0.500). 6MWD = 6-minute walk distance, LVEF = left ventricular ejection fraction, NT-proBNP = plasma NT-pro-brain natriuretic peptide.

When participants were at supine position, the patients in group 1 had slightly higher CO (3.70 ± 0.25 vs 3.14 ± 0.27 L/min), CI (2.04 ± 0.13 vs 1.82 ± 0.14 L/min/m^2^), SV (54.38 ± 3.68 vs 49.70 ± 4.64 mL), SVI (29.92 ± 1.85 vs 28.70 ± 2.37 mL/m^2^), LSW (61.77 ± 4.52 vs 52.49 ± 4.94 gm-m/beat), and LSWI (33.87 ± 2.23 vs 30.13 ± 2.47 gm-m/m^2^/beat) than those parameters in group 2 (Table 6). Whereas SSVR (444.75 ± 30.22 vs 434.69 ± 43.64 dynes/cm^5^) and SSVRI (247.94 ± 17.78 vs 252.81 ± 23.60 dynes/cm^5^/m^2^) in group 1 were slightly higher than those parameters in group 2. NT-proBNP (172.92 ± 62.79 vs 431.13 ± 95.46 pg/mL; *P* = 0.059) and LVEF (64.04 ± 1.64 vs 64.30 ± 1.46%; *P* = 0.500) had no significant difference in the 2 groups (Fig. 2B and C, Table 6). Whereas patients in group 1 had significantly higher 6MWD than patients in group 2 (515.38 ± 24.97 vs 306.39 ± 20.20 m; *P* = 0.043) (Fig. 2A, Table 6).

**Table 6:**
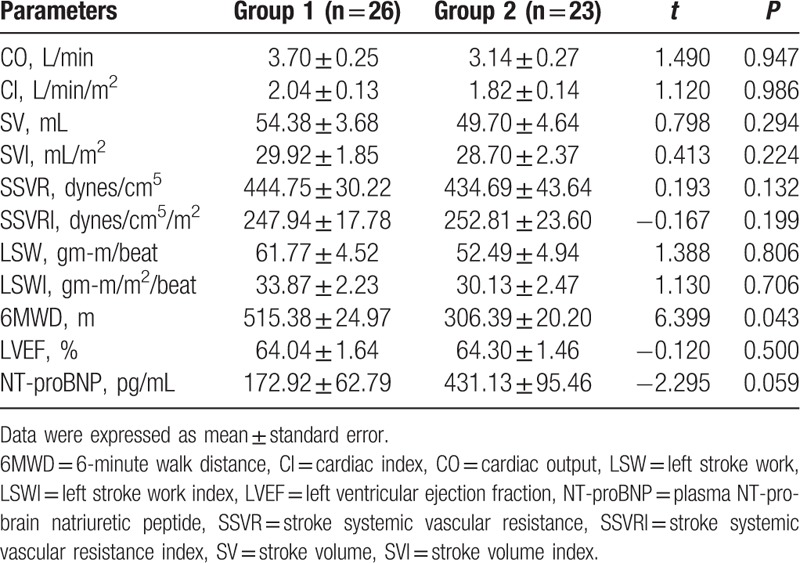
Comparisons of parameters at supine position between group 1 and group 2.

## 4 Discussion

In previous study, ICG has been used to reveal hemodynamic characteristics in heart failure.^[32]^ However, few studies have been published on the hemodynamic changes in response to PLR in patients with HFPEF. Our study found that after PLR, the hemodynamic parameters of some patients rose and others did not rise. We separated the subjects into 2 groups: the patients whose hemodynamic parameters rose after PLR were in group 1 (n = 26) and the patients whose hemodynamic parameters did not rise after PLR were in group 2 (n = 23).

The 2 groups had similar cardiac structure according to echocardiography parameters LAD, DLVD, IVST, and LVPWT (Table 1). The parameter E/E′ (8.85 ± 0.63 vs 10.26 ± 0.65; *P* = 0.540) in echocardiography was not significantly different between the 2 groups. Early-to-late mitral filling velocity ratio (E/A) (0.90 ± 0.06 vs 0.77 ± 0.04; *P* = 0.045) was significantly different between the 2 groups. E/A ratio usually decreased to be less than 1 in patients with diastolic dysfunction.^[33,34]^ A higher E/A ratio in group 1 likely represents a less degree of diastolic stiffness, and therefore a better cardiac preload reserve,^[35]^ which in itself would predict a better response to 6MWT. The present study showed that group 1 had significantly higher 6MWD than group 2 (515.38 ± 24.97 vs 306.39 ± 20.20 m; *P* = 0.043). As the role of 6MWD in assessing exercise tolerance and functional capacity in patients with impaired cardiac function,^[36]^ our study indicated that the patients in group 1 had better cardiac function and exercise capacity than patients in group 2.

Our study found that CO, CI, SV, SVI, LSW, and LSWI increased, and SSVR and SSVRI decreased after PLR in group 1; CO, CI, SV, SVI, LSW, and LSWI did not increase, and SSVR and SSVRI increased in group 2 (Tables 2 and 3). According to the curvilinearity of the Frank–Starling relationship, if the heart is operating on the initial and steep part of the curve, it should have some preload reserve, and an increase in cardiac preload results in an increase in SV.^[18,37]^ Previous studies of hemodynamic effects of PLR indicated that hemodynamic changes related to PLR were only induced by increased cardiac preload.^[38]^ CO in response to PLR was according to the central volume status and the degree of cardiac preload reserve.^[39]^ Our study indicated that patients in group 1 had better central volume status and larger cardiac preload reserve than patients in group 2.

Previous studies showed that LVEF and NT-proBNP levels were correlated with cardiac function.^[40,41]^ Even though NT-proBNP (172.92 ± 62.79 vs 431.13 ± 95.46 pg/ml; *P* = 0.059) had no significant difference between the 2 groups, NT-proBNP significantly inversely correlated with 6MWD (*R* = −0.539, *P* < 0.001). The present study showed that there was no significant difference in the hemodynamic parameters measured by ICG between the 2 groups. Consistent with the hemodynamic parameters, our study found that LVEF (64.04 ± 1.64 vs 64.30 ± 1.46%; *P* = 0.500) was not significantly different between the 2 groups. Our results also showed that LVEF was not correlated with 6MWD (*R* = −0.083, *P* = 0.570).

Our study found that CO, CI, SV, SVI, LSWI, and LSW correlated positively with 6MWD, whereas SSVR and SSVRI correlated negatively with 6MWD in 2 groups (Table 4). With higher CO, CI, SV, SVI, LSWI, and LSW, patients would have stronger functional capacity, exercise tolerance, and longer 6MWD. Our study may indicate that the patients in group 1 whose hemodynamic parameters (CO, CI, SV, SVI, LSWI, and LSW) rose after PLR, had better exercise capacity and cardiac function. The hemodynamic variation after PLR could screen patients with cardiac dysfunction, which would contribute to better management of HFPEF. Digoxin therapy could increase the CO, improve exercise capacity, and reduce symptoms in patients with HFPEF.^[42]^

Our study has limitations. The subjects in our study were relatively older and their parameters maybe could not represent the state of the whole population. The sample of the study was also small, which limited the statistical power of group analyses.

## 5 Conclusions

Measuring hemodynamic parameters by ICG, the patients whose CO, CI, SV, SVI, LSW, and LSWI increased, whereas SSVR and SSVRI decreased in response to PLR, were more likely to have better exercise capacity. Hemodynamic variation in response to PLR measured by ICG may be sensitive in predicting exercise capacity of patients with HFPEF.

## Acknowledgments

The authors are thankful for the vital contribution of the participating volunteer patients in this study. The authors would like to be grateful to the local research ethics committee and the individual researchers, clinicians, and nurses who participated in the study.
